# Physical exercise as add-on treatment in adults with ADHD – the START study: a randomized controlled trial

**DOI:** 10.3389/fpsyt.2025.1690216

**Published:** 2025-10-30

**Authors:** Lena Axelsson Svedell, Mialinn Arvidsson Lindvall, Kajsa Lidström Holmqvist, Yang Cao, Mussie Msghina

**Affiliations:** ^1^ Department of Psychiatry, Faculty of Medicine and Health, Örebro University, Örebro, Sweden; ^2^ School of Health, Care and Social Welfare, Mälardalen University, Västerås, Sweden; ^3^ Department of Neurology and Rehabilitation Medicine and University Health Care Research Center, Faculty of Medicine and Health, Örebro University, Örebro, Sweden; ^4^ Clinical Epidemiology and Biostatistics, School of Medical Sciences, Faculty of Medicine and Health, Örebro University, Örebro, Sweden; ^5^ Unit of Integrative Epidemiology, Institute of Environmental Medicine, Karolinska Institute, Stockholm, Sweden; ^6^ Department of Clinical Neuroscience, Karolinska Institute, Stockholm, Sweden

**Keywords:** physiotherapy, non-pharmacological, quality of life, insomnia, intervention

## Abstract

Adult ADHD is associated with various health challenges and reduced quality of life. Current guidelines recommend multimodal treatment, and physical exercise has emerged as a promising non-pharmacological alternative, although evidence from randomized controlled trials remains limited. In this randomized controlled trial, we aimed to assess the clinical effectiveness of physical exercise as an add-on treatment for adults with ADHD compared to treatment as usual. The trial included adults with a clinically confirmed diagnosis of ADHD was conducted at one Psychiatric clinic in Sweden. Participants were randomly assigned (2:1, no stratification) using an electronic case-report platform, to either physical exercise (the protocolized 12-week intervention START) or treatment as usual (local community care). Primary outcome was the ASRS-v1.1 Symptom checklist at 12 weeks after inclusion. The analysis followed a modified intention-to-treat principle, excluding participants who provided no data beyond baseline. Of the 63 participants enrolled, 43 were randomly assigned to START physical exercise intervention and 20 to treatment as usual. After accounting for withdrawals (n = 11) and loss to follow up (n = 11), the primary analysis included data from 41 participants (30 assigned to START intervention and 11 to treatment as usual). The START intervention resulted in improved ADHD symptoms after 12 weeks, as measured by ASRS-v1.1. Symptom improvement differed significantly between groups (mean difference -6.98, 95% CI: -12.30 to -1.65; p = 0.012) with an effect size of 0.93 favoring the intervention group. No serious adverse events were reported. The results suggest that physical exercise may be a feasible, safe and clinically meaningful complement to standard care for adults with ADHD. However, the findings should be interpreted in the light of potential confounders and methodological limitations. This trial is registered with the ClinicalTrials.gov. Date of registration: 2021-05-14.

**Clinical trial registration:**

https://clinicaltrials.gov/study/NCT05049239, identifier NCT05049239.

## 1 Introduction

The prevalence of attention deficit hyperactivity disorder (ADHD) is about 2.5% among adults (1). Beyond the core symptoms of inattention, impulsivity, and hyperactivity individuals with ADHD often experience additional health challenges. These include somatic conditions such as cardiovascular disease and obesity, as well as psychiatric comorbidities including substance use disorder, depression and anxiety (2). Sleep problems are also highly prevalent and associated with the psychiatric comorbidities (3). Furthermore, adults with ADHD frequently report a reduced quality of life (2), struggle in social and professional settings, and face an increased risk of unemployment or long-term sick leave (4). These factors contribute to significant health disparities and highlight the impact ADHD has on daily functioning (5).

Current research and treatment guidelines (6, 7) recommend a multimodal treatment approach that combines pharmacological and non-pharmacological interventions. While central stimulants remain the mainstay of treatment, they have notable limitations. Although effective in the short term, their long-term efficacy remains uncertain (7, 8) and the treatment may carry risks for cardiovascular side effects (7) and potential for abuse. Additionally, approximately 30% of patients respond poorly to pharmacotherapy (9) and even when symptom relief is achieved, physiological and psychological side effects often lead to treatment discontinuation (10). These limitations underscore the need for alternative, better tolerated, non-pharmacological treatment options that can effectively manage ADHD symptoms and associated health challenges (6, 7, 11).

Regular physical exercise has emerged as a promising non-pharmacological treatment alternative for ADHD (12, 13). In the general population, regular physical activity has well-documented benefits for both physical and mental health, including improved cognitive function, mood, and overall physical health (14). In children and adolescents with ADHD, structured physical exercise programs have been shown to improve core symptoms of ADHD and cognitive function (15). However, despite these promising findings, evidence regarding the benefits of structured physical exercise for adults with ADHD remains yet to be established (16). Given that individuals with ADHD face unique executive challenges that affect their goal-directed behavior (17), the development and evaluation of a physical exercise intervention specifically tailored to this population is both timely and necessary.

To address this unmet need, we developed the START intervention (START = Stöd i Aktivitet, Rörelse och Träning [Support in Activity, Movement, and Exercise]) a structured, physiotherapist-led, moderate-intensity mixed exercise program, delivered as an add-on to standard care. The intervention is designed to increase internal motivation and address challenges with goal-directed behavior often associated with ADHD (18), through supervised sessions offering flexibility via individual coaching and additional exercise opportunities. By an interval-based format, combining endurance, strength, and flexibility training, it allows for individualized intensity adjustments (19). Targeting both ADHD symptoms and physical fitness, the intervention aims to reduce symptoms and also promote health in adults with ADHD.

A pilot study was conducted to assess the feasibility and tolerability of the START intervention, with findings indicating promising trends in ADHD symptom improvement and enhanced well-being (20). If proven effective, the START intervention could become a valuable addition to current treatment options. In this article, we report the findings of the START study, a randomized controlled trial (RCT) based on the hypothesis that regular, structured physical exercise can complement standard ADHD care and improve function and quality of life (QoL) for adults with ADHD. This study aims to evaluate the effect of regular, structured physical exercise as an add-on treatment for ADHD symptoms, insomnia and health related QoL in adults with ADHD, compared to treatment as usual (TAU).

## 2 Materials and methods

### 2.1 Study design

The START study is a parallel randomized controlled trial (RCT) designed to assess the clinical effectiveness of regular, structured physical exercise as add-on treatment for adults with ADHD, compared to TAU. The study was conducted in a medium-sized Swedish County at one open care psychiatric clinic that is part of the Swedish National Health Service. Ethics approval was obtained from the Swedish Ethical Review Authority, reference number EPM 2020–05641 and 2021–02743 on December 18, 2020. The trial was registered at the Clinical Trials.gov with the identifier NCT05049239. The study protocol has been published previously (19) and can be found on https://bmcpsychiatry.biomedcentral.com/articles/10.1186/s12888-023-05181-1.

### 2.2 Participants

Participant recruitment took place from Feb 23, 2021, to Feb 21, 2024. Study participants were adults (18 to 65 years) with a confirmed ADHD diagnosis based on Swedish national guidelines. The ADHD diagnosis was established before inclusion in the study through an extensive neuropsychological evaluation by a specialized team of psychiatrists and psychologists in accordance with DSM 5 diagnostic criteria. Eligibility for participation and enrolment was assessed by a consultant psychiatrist and a physiotherapist, both members of the research team. The exclusion criteria were: 1) a physical activity level exceeding 150 minutes per week, 2) severe depression at the time of enrolment, 3) a history of suicidal behavior, 4) active ongoing addiction, or 5) a severe psychiatric disease such as psychosis and bipolar disease, or severe autism. The threshold of 150 minutes per week was chosen based on the World Health Organization’s recommendations for minimum levels of physical activity required for health benefits. Individuals already exceeding this level were excluded to avoid potential ceiling effects. Sex data were reported by participants with options female or male. All prospective participants provided written informed consent. If the confirmed diagnosis was missing or the recruiting psychiatrist deemed the individual to have severe psychiatric comorbidity which would interfere with their ability to participate in the START intervention, they were not considered for inclusion in the study. Eligibility was ultimately a clinical decision made by the psychiatrist or physiotherapist at the initial face-to-face meeting, rather than by cut-off scores from standardized assessment tools.

Main recruitment was conducted from an open care General Psychiatry clinic and a Clinic for Young Adults at a university hospital in Sweden. Participants were also recruited via the following channels: the 1177 website (the official website for all county-based health care in Sweden), the Örebro County Facebook account, and the National Association for Neurodevelopmental Disorders, Riksförbundet Attention.

### 2.3 Randomization and masking

After enrolment, the trial coordinator (LAS) randomly assigned participants (2:1) to either the START physical exercise intervention or to TAU using the pre-programmed randomization module in the electronic case report platform (e-CRF) Greenlight Guru Clinical, hosted by Region Örebro County (Örebro, Sweden). The randomization sequence was computer-generated and programmed by an independent statistician, who had no further involvement in the trial. Block randomization with random block sizes of 2, 4, and 6 ensured even allocation between groups. No stratification was applied.

The trial coordinator participated in data collection (Clinical Global Impression-Severity scale (CGI-S) and Clinical Global Impression-Improvement scale (CGI-I) and data analysis and handled both enrolment and assignment to the study groups. The statistician involved in data analysis remained blinded to treatment allocation. However, due to the nature of the intervention, it was not possible to blind the participants or the clinicians administering the intervention.

### 2.4 Procedures

A standardized enrolment process was implemented to ensure participant eligibility and adherence to study procedures. At first, before enrolment in the trial, a consultation was conducted by primary investigator (MMS) and the trial coordinator (LAS). During this appointment, individuals were given oral and written information about the study and the procedures. If the individual met the eligibility criteria, and did not have any exclusion criteria or was not clinically assessed to have interfering psychiatric comorbidity, they were asked for consent to participate. After obtaining oral consent, written informed content was collected, and the participants’ medical records were reviewed to confirm the ADHD diagnosis. Following the initial appointment, the randomization was conducted and a link to the self-report questionnaires was sent to the participants’ e-mail including a reminder text message to their smartphone. All self-report questionnaires were completed digitally on the eCRF Greenlight Guru Clinical. In addition to the digital self-report, a face-to-face appointment was scheduled with a licensed physiotherapist from a team at the Unit for Psychiatric Physiotherapy in Region Örebro County. During this second meeting, the physiotherapist informed the participant about their assigned intervention and explained the tasks involved. All clinical data were collected at one Psychiatric clinic at Örebro University Hospital in Örebro, Sweden.

The START intervention was a protocol-based, physical exercise program designed to adapt to individual conditions (19) and to increase internal motivation. The program consisted of a 12-week moderate-intensity mixed physical exercise that included focus on bodily sensations to improve body awareness and cognitive skills training. It involved supervised interval-based sessions combining endurance, strength, and flexibility training as well as self-selected physical activities. Participants were encouraged to attend at least two 50-minute supervised sessions per week, each led by two licensed physiotherapists experienced in psychiatry. Each session began with a brief self-assessment of how the participant felt that day, including bodily sensations, followed by a warm-up, interval-based strength and aerobic training, and concluded with a cool-down including stretching. The supervised sessions were structured regarding interval duration, exercise selection and order, and targeted 60-90% of maximum heart rate, monitored via Polar H10 heart rate monitors. In addition to the supervised sessions, participants were encouraged to engage in additional physical exercise outside the clinic, aiming for at least 150 minutes per week within the target heart rate (HR) zone (> 60% of maximum HR). Throughout the intervention, participants were encouraged to stay mindful of their own body and to reflect on how their body felt while performing the exercise. At least one physiotherapist was always close by, ready to reassure the participant and explain any unfamiliar or frightening bodily reactions that could occur during the session. In the START intervention, a subset of participants (n=11 of 43) were offered cognitive skills training to improve adherence to protocol. This training focused on self-prioritized goals related to time-management, planning and organization in everyday life, with examples including to arrive on time for training or to create the space for physical activity. However, for the purposes of this study, all participants who received the START intervention, regardless of whether they received cognitive skills training or not, were considered part of the START intervention group. Participants continued with any ongoing pharmacological and/or nonpharmacological treatment, other than structured physical exercise.

The comparator condition was treatment as usual (TAU), as defined previously. Due to the pragmatic nature of the trial no attempt was made to standardize the content of TAU.

The intervention period was 12 weeks for both groups. Assessments were done remotely at week 6 using online forms, including the WHO Adult ADHD Self-Report Scale Symptom checklist (ASRS-v1.1) (21), the Patient-rated Global Impression-Improvement scale (PGI-I) (22), the Insomnia Severity Index scale (ISI) (23) and EuroQol visual analogue scale (EQ VAS) (24). 12-week assessments were done within three weeks after the end of the intervention, and conducted both remotely with the same online forms (ASRS-v1.1, PGI-I, ISI and EQ VAS) and face-to-face, using the CGI-I and the CGI-S (22). The methodology and feasibility of the intervention have been comprehensively described in articles previously published by the research group (19, 20).

### 2.5 Outcome measures

The primary outcome was self-reported ADHD symptoms measured with the ASRS-v1.1 Symptom checklist (21). The ASRS-v1.1 Symptom checklist is a symptom profile and symptom burden scale for adult ADHD that consists of 18 questions corresponding to the 18 criteria for ADHD according to the Diagnostic and Statistical Manual of Mental Disorders, Text Revision, 4th edition. The participant reports their ADHD-symptoms on the rating scale: 0 = never, 1 = rarely, 2 = sometimes, 3 = often and 4 = very often. Scores range from 0 to 72, with the maximum score of 72 indicating maximum symptom burden. The ASRS-v1.1 has shown good reliability and validity (25) regardless of if the report is done by the patient him/herself or administered by a clinician. The total score of the ASRS-v1.1 was used in this study as it corresponds to symptom burden and is commonly used for treatment evaluation.

Several secondary outcome measures of clinical effectiveness were used. The CGI-S (22) is measured on a 7-point Likert scale and reflects the patient’s total psychiatric impairment. It provides a clinician-rated measure that considers all available information, including knowledge of the person’s history, psychosocial circumstances, symptoms, and behavior, and the impact of the symptoms on the person’s ability to function. The clinician rates severity of illness in answer to the question “Considering your total clinical experience with this particular population, how mentally ill is the patient at this time?”. The responses are ranged from 1-7, with 1 = normal, not at all ill, 2 = borderline mentally ill, 3 = mildly ill, 4 = moderately ill, 5 = markedly ill, 6 = severely ill and 7 = among the most extremely ill of subjects (22).

The Clinical Global Impression-Improvement scale (CGI-I) (22) is measured on a 7-point Likert scale and reflects improvement and treatment satisfaction. The clinician rates the patient’s improvement in answer to the question “Compared to the subject´s condition at baseline, how much has he/she changed?” with the responses from 1 = very much improved, 2 = much improved, 3 = minimally improved, 4 = no change, 5 = minimally worse, 6 = much worse and 7 = very much worse (22).

The Patient-rated Global Impression-Improvement scale (PGI-I) is self-rated, measured on the same 7-point Likert scale as CGI-I (22).

Insomnia Severity Index (ISI) (23) scale is a self-report questionnaire with seven items assessing the nature, severity and impact of insomnia. Each item is rated on a 0–4 scale and the total score ranges from 0 to 28. A higher score suggests more severe insomnia. The ISI scale has been reported to have good reliability and validity (23).

The EQ VAS (24) is a well-established health-related QoL self-report instrument for self-assessed health status, developed by the EuroQol Group. It consists of a visual analogue scale (VAS) from 0 to 100 with the endpoints labelled “Worst imaginable heath state” (0) to “Best imaginable health state” (100). The individual indicates the point on the scale that represents their current health state (24). The EQ VAS has been found useful to evaluate healthcare performance and health in a Swedish population. The VAS value for full health is 88.9 in the Swedish value set and the dimension for anxiety/depression has the greatest impact on VAS outcomes (26).

To monitor the safety of the intervention, harms were defined as any physical or psychological adverse effect or injury potentially attributable to the exercise program. Data on harms were collected systematically during supervised sessions by physiotherapists and continuously through participant-initiated reporting. All adverse events were recorded in the study’s eCRF system.

### 2.6 Statistical analysis

The target sample size was calculated based on the primary outcome: reduction in ADHD symptoms assessed with the ASRS-v1.1 Symptom checklist. A mean difference in symptom reduction of 15% between the intervention and control groups was assumed, with a standard deviation of 3%, a two-sided alpha of 0.05, and a power of 0.8. Anticipating a 25% drop-out rate, the required sample size was estimate at 75 participants, corresponding to 63 (= 75 x 75%) participants needed for the expected power of 0.8.

Descriptive statistics were calculated for all variables of interest. Continuous variables are reported as means ± standard deviations, and categorical variables as counts and percentages. The 7-point CGI-I and PGI-I scales were dichotomized into two categories: Clinically meaningful improvement (“very much improved” or “much improved”) and not improved (“minimally improved”, “no change”, “minimally worse”, “much worse” or “very much worse”) for analysis.

Comparisons of baseline demographic and clinical characteristics between the 2 groups were conducted using independent-samples t-tests or Chi-squared tests, as appropriate. Primary analyses followed a modified intention-to-treat (ITT) approach, including all patients who completed at least 1 follow-up after baseline. Missing data were imputed using the “last observation carried forward”-method from the online assessment at week 6. Between-group differences in post-intervention changes were assessed using t-tests, Mann-Whitney U tests, or Chi-squared tests, depending on variable distribution and measurement scale. Effect sizes for continuous outcomes were calculated using Cohen’s d.

To evaluate the robustness of primary outcome findings, a conservative sensitivity analysis was conducted under a strict ITT framework, imputing all missing data as unchanged from baseline to 12 weeks. All analyses were conducted with the statistical software SPSS version 29.0 (IBM Corp., Armonk, NY, USA) and statistical significance was defined as a 2-tailed p-value of < 0.05.

## 3 Results

**Figure 1:**
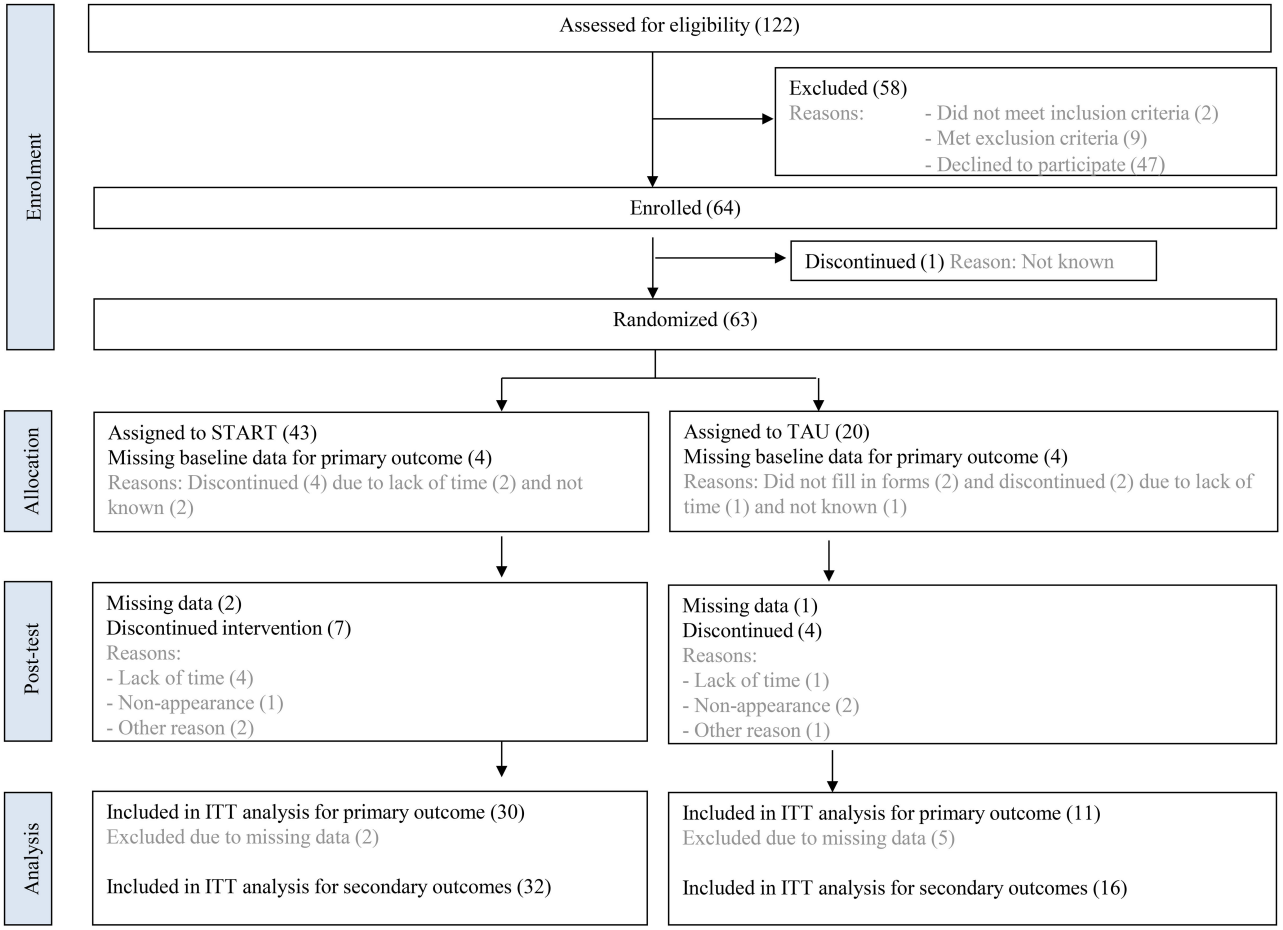
Participant flow diagram with reasons for drop-out. The diagram illustrates the number of individuals screened for eligibility, randomized to the START intervention or TAU group, and completing the 12-week follow-up period. Reasons for drop-out are specified at each stage. START, START physical exercise intervention; TAU, Treatment as usual; ITT, Intention to treat.

One hundred twenty-two individuals were screened for eligibility. Out of these, 47 declined to participate and 12 were ineligible due to; did not meet inclusion criteria (n = 2), met exclusion criteria (n = 9) and did not show up (n = 1). Sixty-three participants were found eligible and were randomly assigned to the START intervention (n = 43) or treatment as usual (TAU) (n = 20). Participant enrolment and a flow chart based on primary outcome is presented in **Figure 1**. Twenty-two participants had missing data at the 12-week assessment due to loss to follow-up (n = 11) or withdrawal (n = 11) (13 in the intervention and 9 in the control group). As a result, complete data for primary outcome was available for 41 participants (65%), while data for any secondary outcome was available for 48 participants (76%) (**Figure 1**).

**Table 1** presents baseline data and clinical characteristics for the intervention group, control group and for the total sample. Forty-two females and 21 men were included in the study with mean age of 36.1 years (SD 9.7), a mean baseline ASRS-v1.1 score of 44.07 and mean CGI-S score of 4. About 2/3 of the participants (63%) had ongoing ADHD medication and about half of the participants (46.4%) reported sedentary behavior of 10 hours or more per day.

**Table 1:** Baseline characteristics of enrolled participants (n=63).

Variables	Total sample (n = 63)	Intervention group (n = 43)	Control group (n = 20)
Sex^a^			
Female	42 (66.7)	31 (72.1)	11 (55)
Male	21 (33.3)	12 (27.9)	9 (45)
Smoking^a^			
Never smoked	34 (54)	25 (58.1)	9 (45)
Former smoker	20 (31.7)	12 (27.9)	8 (40)
Smokes sometimes	5 (7.9)	3 (7.0)	2 (10)
Daily smoker	4 (6.3)	3 (7.0)	1 (5)
Relationship status^a^			
Living alone	25 (39.7)	17 (39.5)	8 (40)
Living with a partner	38 (60.3)	26 (60.5)	12 (60)
Highest educational attainment^a^			
Primary- and lower secondary	5 (7.9)	4 (9.3)	1 (5)
Upper secondary	35 (55.6)	23 (53.5)	12 (60)
Higher	23 (36.5)	16 (37.3)	7 (35)
Ongoing ADHD medication^a^			
Yes	40 (63.5)	27 (62.8)	13 (65)
No	23 (36.5)	16 (32.7)	7 (35)
Sedentary behavior^a^			
10 hours or more	26 (46.4)	18 (46.2)	8 (47.1)
Less than 10 hours	30 (53.6)	21 (53.8)	9 (52.9)
Age in years ^b^	36.13 ± 9.73	35.42 ± 9.48	37.65 ± 10.33
BMI kg/m^2b^	27.22 ± 4.54	26.91 ± 4.76	28.00 ± 3.97
Waist circumference, cm^b^	94.6 ± 12.09	92.46 ± 11.86	100.06 ± 11.25
Resting heart rate, seated^b^	82.87 ± 13.39	81.67 ± 11.91	85.40 ± 16.10
Blood pressure, systolic^b^	126.45 ± 15.96	125.36 ± 15.42	128.75 ± 17.22
Blood pressure, diastolic^b^	84.81 ± 10.65	83.36 ± 9.78	87.85 ± 11.98
MADRS-S^b^	16.57 ± 7.49	16.93 ± 7.42	15.80 ± 7.77
EQ VAS^b^	51.29 ± 20.99	52.11 ± 22.55	49.47 ± 20.18
ASRS-v 1.1 sum^b^	44.07 ± 8.04	44 ± 7.83	44.25 ± 8.78
ASRS-v 1.1 subscale hyperactivity^b^	19.80 ± 5.19	19.97 ± 5.24	19.38 ± 5.20
ASRS-v 1.1 subscale inattention^b^	24.28 ± 4.55	24.03 ± 4.54	24.88 ± 4.66
Physical activity-minutes^c^	105 ± 150	90 ± 191	105 ± 120
CGI-S^c^	4.00 ± 1	4.00 ± 1	4.00 ± 1

^a^ Categorical variable, data are presented as numbers and percentages. ^b^ Normally distributed numerical variable, data are presented as mean ± standard deviations. ^c^ Not normally distributed numerical variable, data are presented as median and interquartile range.

ADHD, Attention Deficit Hyperactivity Disorder; ASRS-v 1.1, The WHO Adult ADHD Self-Report Scale Symptoms checklist v1.1; BMI, Body mass Index; CGI-I, Clinical Global Impression- Severity; EQ VAS, EuroQol Visual analog scale; MADRS-S, Montgomery and Asberg Depression Ration Scale Swedish version.

The primary outcome, ADHD symptoms measured with ASRS-v1.1, differed significantly between intervention and control group at post-intervention (Mean difference: -6.98, 95% CI: -12.30, -1.65, p = 0.012), favoring the intervention group, with a large effect size (Cohen´s d: 0.93). The mean ASRS score decreased by -7.07 (SD: 7.99) points from baseline to 12 weeks in the intervention group and decreased by -0.09 (SD: 5.71) points in the control group (**Table 2**).

**Table 2:** Between-group mean differences and effect sizes at week 12.

Outcome variables	Mean difference (intervention – TAU)	95% CI mean difference	t-value	Degrees of freedom	p-value	Effect size (d) (95% CI)
ADHD symptoms ^a^	-6.98	-12.30, -1.65	-2.65	39	0.012*	-0.93 (CI: -1.65, -0.21)
Insomnia ^a^	-3.84	-1.22, -6.46	-2.97	39	0.005*	-1.05 (CI: -0.31, -1.77)
EQ VAS ^b^	13.00	24.15, 1.86	2.36	38	0.023*	0.58 (CI: 1.28, -0.13)
CGI-I Clinician assessed	2.19	1.57, 2.81	7.13	39	< 0.001*	2.45 (CI: 1.58, 3.30)
PGI-I Patient assessed	1.80	1.20, 2.39	6.10	35	< 0.001*	2.26 (CI: 1.35, 3.15)

Mean difference = (mean change in intervention group) – (mean change in TAU group). ^a^ Negative values indicates greater improvement in intervention group compared to control, ^b^Positive value indicates a greater increase in health-related quality of life in intervention group compared to control, * Two-tailed p < .05 for intervention group compared to control group.

Bold values indicate statistically significant values at p < 0.05.

The intervention group showed significant improvements in all secondary outcomes compared to the control group (**Tables 2**, **3**). For total severity of illness measured with CGI-S, the intervention group showed a mean improvement of -0.61 (SD: 0.737), while the control group showed a mean deterioration of 0.25 (SD: 0.452, p < 0.001). The effect size was large (Cohen´s d: -1.28).

**Table 3:** Between-group difference in Clinician-assessed (CGI-I) and patient-assessed (PGI-I) improvement at 12 weeks.

Outcome variables	Group	Improvement n (%)	No improvement n (%)	X^2^	p-value
CGI-I Clinician assessed	Intervention (n = 29)	19 (65.5)	10 (34.5)		
	Control (n = 12)	0 (0)	12 (100)	14.652	**< 0**.001*
	Total (n = 41)	19 (46.3)	22 (53.7)		
PGI-I Patient assessed	Intervention (n = 27)	12 (44.4)	15 (55.6)		
	Control (n = 10)	0 (0)	10 (100)	6.578	**0**.010*
	Total (n = 37)	12 (32.5)	25 (67.5)		

Data are presented as number of individuals (n) and percentage (%) within each group. ^a^ Improvement was defined as values 1- 2, while no improvement was defined as values 3-7. * Two-tailed p < .05 for intervention group compared to control group. X^2^, Chi-square test.

Bold values indicate statistically significant values at p < 0.05.

Mean scores for clinician- and patient-rated improvement, CGI-I and PGI-I, indicated greater improvement in the intervention group compared with the control group. Intervention group mean score for CGI-I was 2.31 (SD: 0.97) and for PGI-I 2.70 (SD: 0.82) indicating substantial improvement. Control group mean score was 4.50 for both CGI-I (SD: 0.67) and PGI-I (SD: 0.70) somewhere in between “No Change” and “slightly worse” on the CGI-I and PGI-I scale. Between-group differences were significant (p< 0.001) for both measures, with a large effect size (CGI-I ES: 2.25, PGI-I ES: 2.26) (**Table 2**). Participants in the intervention group were significantly more likely to show improvement in CGI-I compared to the control group (RR=9.03, 95% CI: 1.34, 60.8, *p*=0.012). The number needed to treat (NNT) to achieve one additional positive outcome was 1.74 (95% CI: 3.86, 1.13). For self-assessed improvement measured by PGI-I, a similar trend was observed (RR=5.83, 95% CI: 0.79, 36.67, *p*=0.043; NNT=2.74, 95% CI: 21.77, 1.46).

Nineteen participants (65.5%) in the intervention group were classified as having a clinically meaningful improvement based on CGI-I, compared to none in the control group (X^2^ = 14.652, p< 0.001) with a large effect size (Phi = 0.60). Similarly, on the PGI-I, 12 participants (44.4%) in the intervention group reported a clinically meaningful improvement, whereas none in the control group reported a clinically meaningful improvement (X^2^ = 6.578, p = 0.010) The effect size was medium (Phi = 0.42)(**Table 3**).

For health related QoL on EQ VAS, the intervention group improved by a score of 10.55 (SD: 25.77) points, while the control group deteriorated by -2.45 (SD: 9.04) points (p = 0.023), with a medium effect size (Cohen´s d: -0.58).

For insomnia measured with ISI, the intervention group showed a mean improvement of -2.93 (SD: 3.86) while the control group had a mean deterioration of 0.91 (SD: 3.081, p = 0.005) with a large effect size (Cohen´s d: 1.05).

In the intervention group, all assessed outcome measures showed significant improvement post-intervention compared to baseline (p < 0.05 in all cases), with medium to large effect on Cohen´s d for ADHD symptom improvement (0.89), insomnia improvement (0.79) and CGI-S improvement (0.82) and small to medium effect (0.41) on improved health related QoL, indicating clinical meaningful improvements. On the other hand, no significant changes were seen in the control group and all effect sizes were small (0.02 to 0.30). ASRS sub scores for inattention and hyperactivity each showed significant improvement in the intervention group. No significant improvements were observed within the control group. **Table 4** shows differences in ADHD symptom, health-related QoL, insomnia and CGI-S between baseline and 12 weeks after the start of the intervention for each group.

**Table 4:** Within-group mean differences and effect sizes between baseline and 12 weeks.

Outcome variables	Group	Baseline	12 week follow up	Mean difference	95% CI mean difference	t-value	p-value two-sided	Effect size (d)
ADHD symptoms^a^ Total score	Intervention (n = 30)	43.67 ± 7.55	36.60 ± 9.33	-7.07 ± 7.99	-10.05, -4.08	-4.85	**< 0**.001*	-0.89 (CI: -1.30, -0.46)
	Control (n = 11)	46.36 ± 9.30	46.27 ± 11.35	-0.09 ± 5.72	-3.93, 3.75	-0.05	0.959	-0.02 (CI: -0.61, 0.58)
ADHD symptoms^a^, subscore inattention	Intervention (n=24)	23.88 ± 4.35	20.21 ± 4.70	-3.67 ± 4.77	-5.68, -1.65	-3.77	**< 0**.001*	0.77 (CI:1.22, 0.31)
	Control (n=11)	26.00 ± 5.04	25.73 ± 5.88	-0.27 ± 3.23	-2.44, 1.90	-0.28	0.393	0.08 (CI: 0.51, 0.68)
ADHD symptoms^a^, subscore hyperactivity	Intervention (n=24)	20.71 ± 3.97	17.46 ± 4.71	-3.25 ± 4.50	-5.15, -1.35	-3.54	0.002*	0.72 (CI: 0.27, 1.17)
	Control (n=11)	20.36 ± 5.45	20.55 ± 6.88	0.18 ± 3.66	-2.27, 2.64	-0.17	0.872	-0.50 (CI: -0.64, 0.54)
EQ VAS ^b^	Intervention (n = 29)	51.24 ± 21.14	61.79 ± 21.27	10.55 ± 25.8	0.75, 20.35	2.21	**0**.036*	0.41 (CI: 0.03, 0.79)
	Control (n = 11)	51.27 ± 23.85	48.82 ± 21.16	-2.46 ± 9.05	-8.53, 3.62	-0.90	0.389	0.27 (CI: – 0.87, 0.34)
Insomnia^c^	Intervention (n = 29)	13.83 ± 6.39	10.90 ± 6.93	-2.93 ± 3.86	-4.37, -1.49	-4.16	**< 0**.001*	-0.76 (CI: -1.16, -0.35)
	Control (n = 11)	10.55 ± 4.37	11.45 ± 5.57	0.91 ± 3.1	-1.16, 2.97	0.98	0.351	0.30 (CI: -0.32, 0.90)
CGI-S	Intervention (n=28)	4.32 ± 1.06	3.71 ± 0.90	0.61 ± 0.73	-0.54, 0.04	4.36	**< 0**.001*	0.82 (CI: 0.39, 1.25)
	Control (n=12)	4.00 ± 1.04	4.25 ± 1.06	-0.25 ± 0.52	0.32, 0.90	- 1.92	0.082	-0.55 (CI: -1.15, 0.07)

Results are calculated with dependent t-test and presented as mean change ± standard deviation, from baseline. ^a^ Negative value of change indicates a reduction in ADHD symptoms, ^b^ Negative value of change indicates an improvement of insomnia, ^c^ Positive value of change indicates an increase in health-related quality of life, * Two-tailed p < .05 for baseline compared to 12 weeks.

ADHD, Attention Deficit Hyperactivity Disorder; CI, confidence interval.

Bold values indicate statistically significant values at p < 0.05.

CGI-I and PGI-I were highly correlated (Spearman’s r = 0.72, p < 0.001), indicating a high level of agreement between clinician- and patient-rated improvements. No serious adverse events were reported related to the intervention.

All randomly assigned participants with baseline data on ASRS were included in sensitivity analysis under a strict ITT framework (total n = 55, 39 in the START intervention group and 16 in the control group), missing data was imputed as unchanged from baseline to 12 weeks. The result from the sensitivity analysis on ASRS shows that the mean difference (mean difference: -4.89, 95% CI: -1.49, -8.28) between the two groups remained significant (p = 0.006), with a slightly reduced effect size compared to the original analysis (Cohen´s d: 0.71, 95% CI: 0.11, 1.30), suggesting that the primary findings are relatively stable even under conservative assumptions about missing data.

## 4 Discussion

This study is, to the best of our knowledge, the first RCT of regular, structured physical exercise intervention for treatment of adult ADHD. Significant improvement, with a robust effect size, was found for the add-on treatment with physical exercise (START) regarding ADHD symptoms, compared to treatment as usual. The intervention followed a well-defined treatment protocol and is described in a way that allows it to be replicated and used by others.

Although similar studies in adults are scarce, previous findings do indicate that both acute bouts of aerobic exercise and longer interventions involving various forms of physical activity hold promise as complementary treatment for adults with ADHD (12, 13). A single session of physical exercise, particularly aerobic activity, can provide immediate improvements in executive functioning, reaction time and ADHD symptoms, as shown by Mehren and colleagues (27) and LaCount et al. (28). A longer intervention that did not target aerobic fitness, such as the 24-week Pilates study by Kouhbanani and colleagues (29), have also demonstrated improvements in sustained attention and attention switching and indicates that extended intervention periods may yield robust results. While the studies on acute effects evaluated aerobic exercise, the long-term study of Kouhbanani et al. (29) evaluated a non-aerobic intervention and the outcomes were mainly focused on cognitive performance, not on ADHD-symptoms *per se*. In our 12-week clinical trial combining aerobic, strength, and flexibility training, we demonstrate that the START intervention can lead to clinically meaningful reductions in ADHD symptoms. Participants in the START intervention were also significantly more likely to self-report improvement, along with enhanced health related quality of life. By that, this study adds information to the growing evidence-base supporting regular physical exercise as a promising adjunct treatment for adults with ADHD. Additionally, our findings suggest that physical exercise in health care settings is a safe treatment option for this population.

Our findings align well with previous research highlighting the positive association between physical activity and improved sleep in adults with ADHD. The study by Zhu and colleagues (30) showed that adults with ADHD who engaged in at least 150 minutes of moderate-to-vigorous physical activity per week reported significantly fewer sleep difficulties, particularly among men. Similarly, our results indicate that participants who engaged in regular physical exercise experienced less insomnia, suggesting that physical activity may counteract some of the sleep disturbances commonly associated with ADHD. Given that stimulant medications can contribute to sleep problems, our findings reinforce the role of exercise as a valuable, non-pharmacological strategy for promoting better sleep. Future research should further explore potential sex differences, and the optimal intensity and duration of physical activity needed to maximize sleep benefits in this population.

Current research and treatment guidelines recommend a multimodal approach that combines pharmacological and non-pharmacological interventions (6, 7), as central stimulants have uncertain long-term efficacy (8), potential cardiovascular risks (7), and side effects that often lead to discontinuation of the pharmacological treatment (10). Our results reinforce the current guidelines by indicating that physical exercise may serve as a safe add-on treatment that can not only reduce ADHD symptoms and increase quality of life but also contribute to the well-documented prevention of non-communicable diseases, including cardiovascular disease, by regular physical activity.

### 4.1 Limitations

Several limitations should be noted. The small sample size with the main analysis performed on 41 participants limits the generalizability of our findings. The high drop-out rate of 35% may also have introduced selection bias as participants who discontinued could have systematically differed from those who completed the study, for example in motivation or symptom severity, potentially affecting the external validity. In addition, the participants were recruited from a single center in Sweden, reducing the geographical and cultural diversity of the sample, which further may limit external validity. Another limitation of this study is the use of t-tests to assess pre–post changes rather than ANCOVA or mixed-model analyses, which may have offered more rigorous control for baseline differences and within-subject correlations (31, 32).

There was a lack of complete blinding for the clinicians that assessed secondary outcomes. Although the primary outcome of ADHD symptoms was self-assessed and web-administered, minimizing the risk of bias from unblinded raters, the results from secondary outcomes may have been affected by the inability to fully mask participants to the clinicians.

A further limitation of the study is that some participants received cognitive skills training in addition to the exercise intervention. Subgroup analyses was originally intended but, in the end, not feasible due to the small sample size. The cognitive skills training introduces a confounding factor, as it may have contributed to changes in the outcome measures that could not be isolated in the results. Moreover, the lack of standardization and control for additional treatments received in the comparator condition TAU introduces potential confounding and limits the ability to attribute improvements solely to the intervention. At the same time, this design mirrors routine clinical conditions and highlights the pragmatic nature of the trial, thereby enhancing ecological validity.

The COVID-19 pandemic posed several challenges during data collection and intervention period of the study. The START intervention could be continued during the pandemic by relocating the supervised exercise sessions outdoors. This allowed participants to maintain attendance while complying with public health pandemic guidelines regarding physical distancing. However, the pandemic significantly slowed the recruitment rate, and we were unable to reach the target sample size of 75 participants. Instead, the study was completed with a total of 63 participants, which limited statistical power. It is also possible that the pandemic contributed to the relatively high drop-out rate, as participants may have faced additional stressors, health concerns, or logistical barriers limiting their participation in the study. These factors are possible confounders potentially affecting the internal and external validity of the results. The ability to implement the program under these difficult circumstances, however, supports its feasibility and flexibility in real-world clinical settings.

Future research should aim to include larger sample sizes to reach statistical power, adopt a multicenter study design to ensure representativity, try to control for confounders and apply a stricter blinding procedure to reduce the risk of bias.

## 5 Conclusion

This study contributes to the growing evidence base that physical exercise can improve ADHD symptoms and promote health in adults with ADHD. Integrating guided physical exercise into routine clinical practice may provide an accessible approach of improving both symptomatic and functional outcomes for adults with ADHD. Taken together with existing evidence, regular structured mixed physical exercise appears to be a valuable add-on treatment that could be integrated within multimodal treatment strategies of ADHD.

## Data Availability

The datasets presented in this article are not readily available because of current Swedish ethical legislation and the European union GDPR act, however pseudonymized data can be requested as a public document and will have to undergo a confidentiality assessment to determine what can be released. Requests to access the datasets should be directed to dso@regionorebrolan.se.

## Glossary

ADHD: Attention deficit/hyperactivity disorder
ASRS: Adult ADHD Self-Report Scale
BMI: Body mass Index
CGI-I: The Clinical Global Impression-Improvement scale
CGI-S: The Clinical Global Impression-Severity scale
DSM 5: The Diagnostic and Statistical Manual of Mental Disorders, Fifth Edition
e-CRF: Electronic case report platform
EQ VAS: European Quality of Life Visual Analog Scale
ISI: Insomnia Severity Index scale
ITT: Intention-to-treat
IQR: Interquartile range
MADRS-S: Montgomery and Asberg Depression Ration Scale Swedish version
NNT: Numbers needed to treat
PGI-I: The Patient-rated Global Impression-Improvement scale
QoL: Quality of life
RCT: Randomized controlled trial
RÖL: Region Örebro county
SD: Standard deviation
START: acronym for “*Stöd i Aktivitet, Rörelse och Träning*” (Support in activity, movement and exercise)
TAU: Treatment as usual
WHO: World Health Organization
